# Typhoid Fever

**Published:** 1875-04

**Authors:** 


Typhoid Fever.—In the *Practitioner* for January Dr. George Johnson says that in the treatment of typhoid fever careful nursing and feeding are of primary importance, while, as a rule, no medicines of any kind are required, and when not required they they are often worse than useless. Diarrhœa is a less frequent symptom than before this plan was adopted, and when it does occur it is far more tractable, while tympanitic distension of the abdomen is a rare event. The mischievous opiate enemata and the torturing turpentine stupes have disappeared together. He believes that one of the main reasons why we have less diarrhœa than formerly is, that we carefully abstain from the employment of irritating drugs of all kinds. As a rule, the fever patient at King's has the "yellow mixture," which is simply colored water, and, except an occasional dose of chloral to procure sleep, and a tonic during convalescence, no active medicines of any kind. These patients are fed mainly with milk, with the addition of beef-tea and two raw eggs in the twenty-four hours, and wine or brandy, varying in quantity according to the urgency of the symptoms of exhaustion, especially in the advanced stages of the disease; but in many of the milder cases, and especially in the case of children, no alcoholic stimulants are required from the beginning to the end of the fever, and when not required they are of course, as says Dr. J., best withheld. He gives no irritating drugs of any kind, and has no doubt that the comparative infrequency of severe and obstinate diarrhœa among his entire fever patients during the last few years is partly attributable to the discontinuance of mineral acid treatment.—*Clinic.*

